# Supplementary figures and images for: Demonstration of a fast and easy sample-to-answer protocol for tuberculosis screening in point-of-care settings: A proof of concept study
Source: PLoS One. 2020 Dec 14;15(12):e0242408. doi: 10.1371/journal.pone.0242408 (PMC7735633; doi:10.1371/journal.pone.0242408)

A

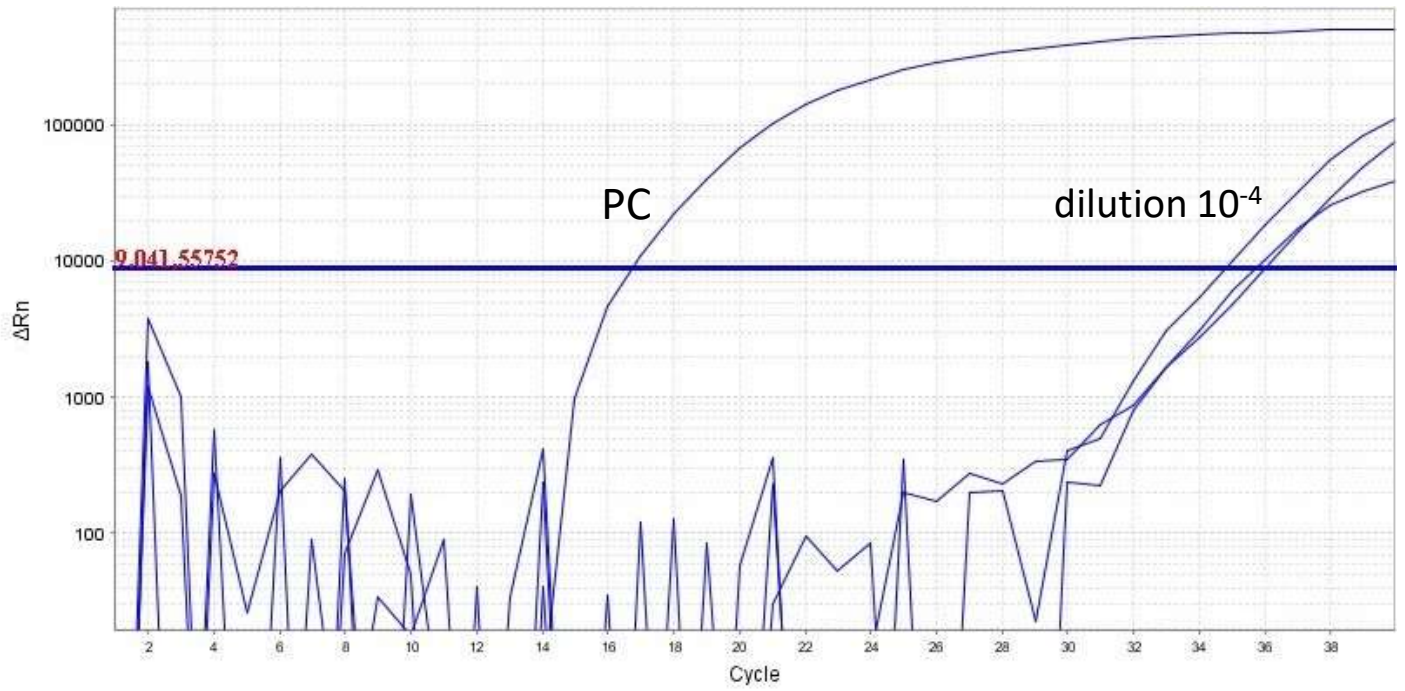

B

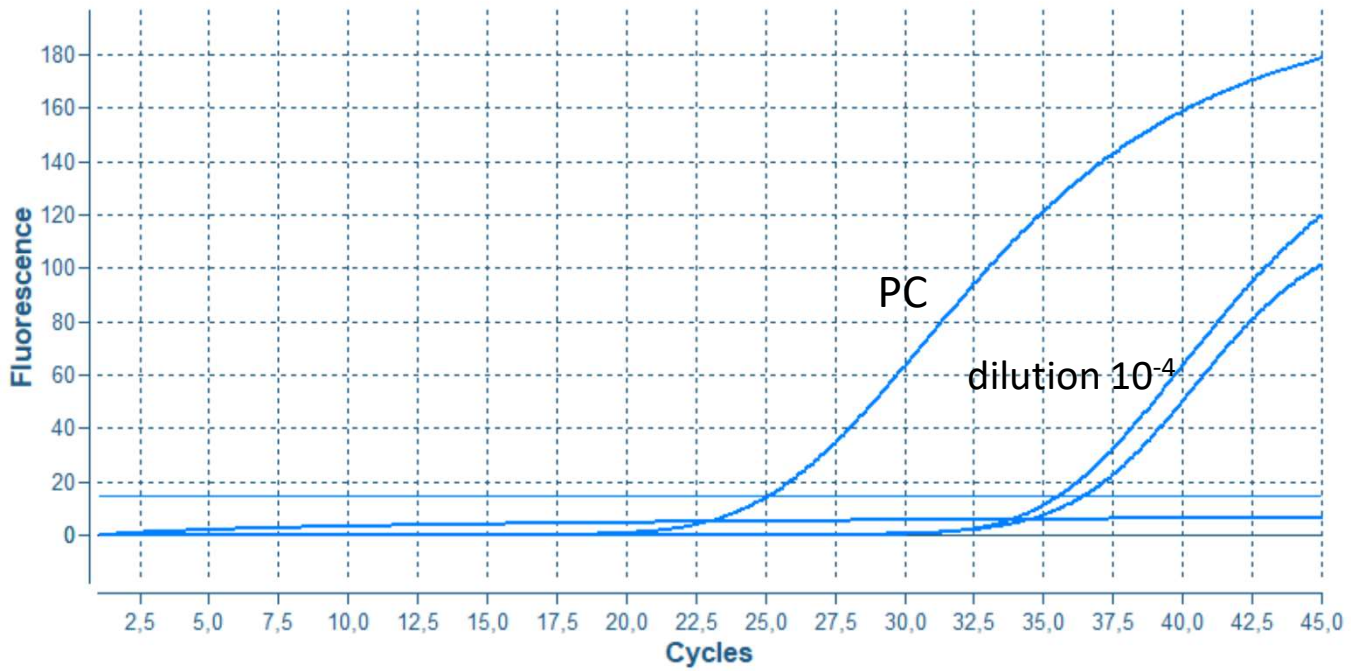

Supplement: S1 Fig — Panel A, trace obtained on the standard ABI7500 with DNA from dilution 10−4. Panel B, trace obtained on the portable Q3-Plus with DNA from dilution 10−4. (PDF) [file pone.0242408.s001.pdf]

A

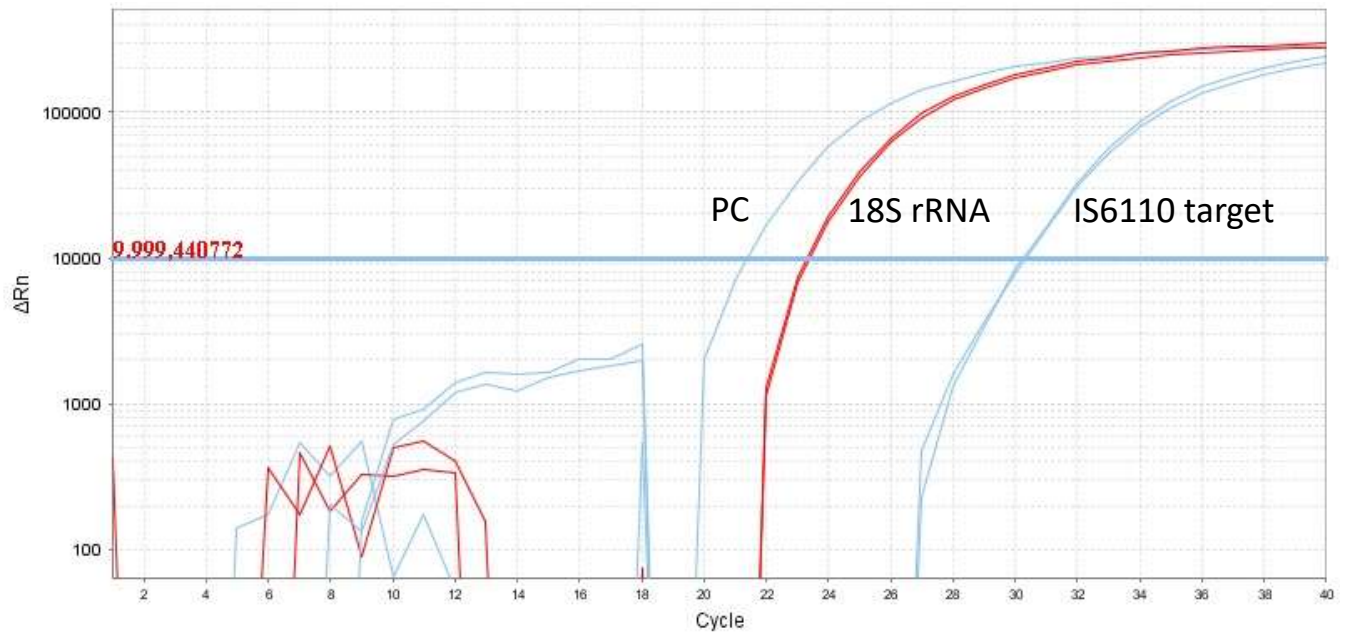

B

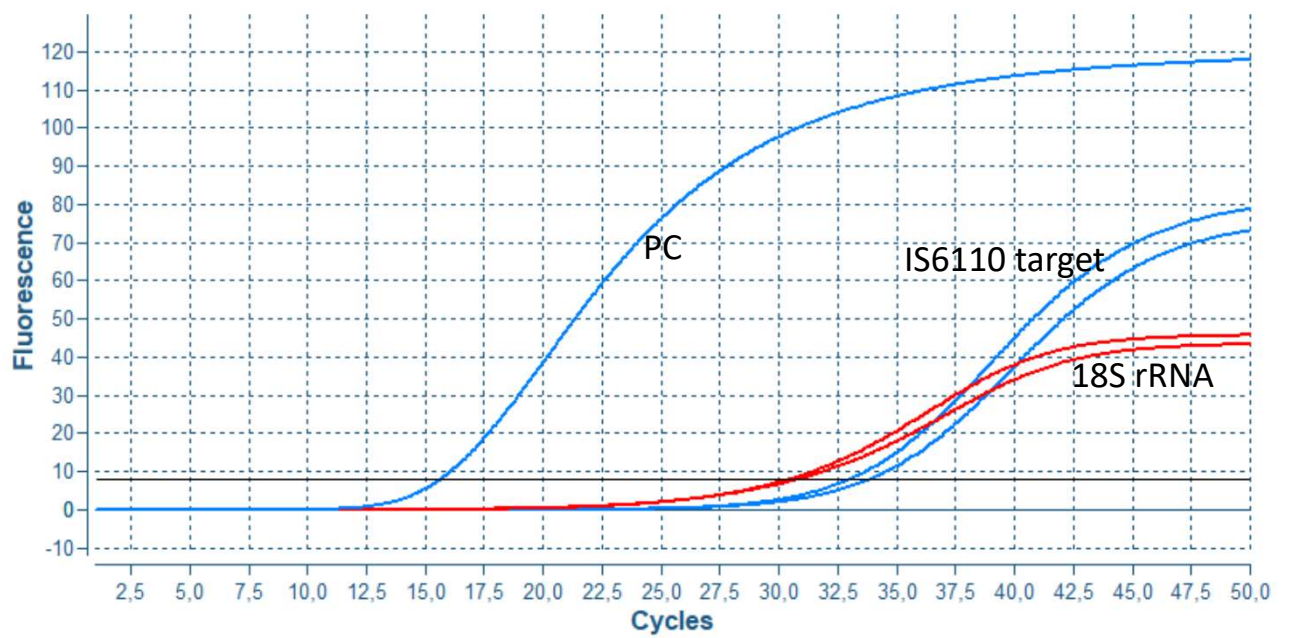

Supplement: S2 Fig — Shown is the detection of the positive control (PC) or the IS6110 target (blue lines) and the human 18S target (red lines). Panel A, trace obtained on the standard ABI7500. Panel B, trace obtained on the portable Q3-Plus. (PDF) [file pone.0242408.s002.pdf]
