# Supplementary material for: Demonstration of a fast and easy sample-to-answer protocol for tuberculosis screening in point-of-care settings: A proof of concept study
Source: PLoS One. 2020 Dec 14;15(12):e0242408. doi: 10.1371/journal.pone.0242408 (PMC7735633; doi:10.1371/journal.pone.0242408)
Supplement: S1 Table — (DOCX) [file pone.0242408.s003.docx]

**S1 Table.** Data used to calculate the averages presented on Table 4.

| Protocol | Dilution | ABI7500 – Regular | | | Q3-Plus - Regular | | | Q3-Plus - Gelified | | |
| --- | --- | --- | --- | --- | --- | --- | --- | --- | --- | --- |
| # 3 | No dilution | ND | | | ND | | | ND | | |
|  | 1:10 | 22.4 | 23.1 | 22.9 | 22.2 | 21.5 | 21.7 | 24.5 | 25.2 | 25.9 |
|  |  | 22.6 | 22.1 | 22.5 | 22.4 | 21.9 | 22.9 | 24.1 | 23.9 | 23.4 |
|  | 1:100 | 25.9 | 25.3 | 26.1 | 25.7 | 25.2 | 24.9 | 29.1 | 27.9 | 28.3 |
|  |  | 25.1 | 26.4 | 24.9 | 24.7 | 26.2 | 25.9 | 28.9 | 27.5 | 29.4 |
| # 4 | No dilution | ND | | | ND | | | ND | | |
|  | 1:10 | 25.2 | 23.8 | 24.6 | 25.4 | 23.7 | 24.5 | 29.5 | 27.8 | 29.9 |
|  |  | 24.2 | 24.9 | 24.0 | 24.1 | 25.1 | 24.6 | 27.1 | 29.4 | 28.9 |
|  | 1:100 | 28.2 | 28.7 | 27.5 | 29.2 | 28.9 | 27.6 | 35.7 | 33.2 | 32.9 |
|  |  | 27.6 | 28.5 | 28.6 | 27.3 | 29.5 | 28.7 | 34.6 | 35.5 | 34.1 |

ND = not detectable
