# Supplementary material for: Demonstration of a fast and easy sample-to-answer protocol for tuberculosis screening in point-of-care settings: A proof of concept study
Source: PLoS One. 2020 Dec 14;15(12):e0242408. doi: 10.1371/journal.pone.0242408 (PMC7735633; doi:10.1371/journal.pone.0242408)
Supplement: S2 Table — (DOCX) [file pone.0242408.s004.docx]

**S2 Table.** Data used to calculate the averages presented on Table 5.

| Dilution | CFU | | ABI7500 | | | Q3-Plus | | |
| --- | --- | --- | --- | --- | --- | --- | --- | --- |
| 10^-1^ | UNC | UNC | 23.5 | 25.2 | 23.9 | 27.5 | 28.2 | 26.8 |
|  |  |  | 24.4 | 23.1 | 24.7 | 28.1 | 27.5 | 26.6 |
| 10^-2^ | UNC | UNC | 26.6 | 27.6 | 28.4 | 30.5 | 31.1 | 29.8 |
|  |  |  | 27.2 | 28.1 | 26.3 | 31.3 | 30.9 | 30.9 |
| 10^-3^ | 14 | 17 | 30.5 | 29.0 | 29.9 | 33.2 | 32.9 | 34.1 |
|  |  |  | 30.7 | 28.9 | 31.1 | 33.9 | 34.6 | 35.1 |
| 10^-4^ | 1 | 2 | 32.5 | 33.7 | 34.3 | 36.7 | 34.8 | 35.5 |
|  |  |  | 33.1 | 34.1 | 32.9 | 34.5 | 36.2 | 35.9 |
| 10^-5^ | NG | NG | ND | ND | ND | ND | ND | ND |

UNC = uncountable

NG = no growth

ND = not detectable
